# Effects of feeding bergamot pulp and olive leaves on performance and meat quality in Apulo-Calabrese pigs

**DOI:** 10.1016/j.vas.2024.100336

**Published:** 2024-01-21

**Authors:** Manuel Scerra, Francesco Foti, Pasquale Caparra, Caterina Cilione, Matteo Bognanno, Fortugno Paolo, De Caria Paolo, Antonio Natalello, Martino Musati, Luigi Chies

**Affiliations:** a University of Reggio Calabria, Dipartimento di Agraria, Produzioni Animali, Via dell'Università, 25, 89124, Reggio Calabria, Italy; b University of Catania, Dipartimento di Agricoltura, Alimentazione e Ambiente (Di3A), Via Valdisavoia 5, 95123, Catania, Italy

**Keywords:** By-product, Phenols, Fatty acids, Tocopherols, Oxidative stability

## Abstract

To determine the effects of dietary inclusion of bergamot pulp and olive leaves on pork quality, thirty-six barrows (Apulo-Calabrese; 112.5 ± 7.40 kg initial bodyweight) were randomly assigned to four experimental treatments and fed for 100 days a control diet (control group) or a diet similar to the control diet in which part of the cereals were replaced with 20 % (DM on the diet fed) of ensiled bergamot pulp (dry matter on the diet fed, EBP group) or olive leaves (OLL group) or a 1:1 mixture of both by-products (BPOL group).

In the present study, the dietary administration of by-products did not influence the accumulation of IMF (*P* > 0.05) in meat. As for individual fatty acids, no differences were observed among groups. However, the dietary supplementation of bergamot pulp tended to increase (*P* = 0.073) the level of α-linolenic acid in meat. In raw and cooked meat TBARS values were higher (*P* < 0.001) in the Control group than in EBP, OLL and BPOL groups. The integration of 20 % of bergamot pulp and/or olive leaves by-products increased the levels of tocopherol and phenolic compounds in the diets, positively influencing the shelf life of meat. No effect of diet was observed on animal performance. The results of present study indicated mainly that partial replacement of cereals with 20 % DM on the diet fed of ensiled bergamot pulp and olive leaves, either alone or in combination, in the pig's diet led to greater oxidative stability of the meat.

## Highlights

• Feeding pigs with bergamot pulp does not negatively affect meat quality.
• Both bergamot pulp and olive leaves increased the levels of phenols in the diet.
• Meat fatty acid composition is not affected by the tested by-products.
• A mixture 1:1 of bergamot pulp and olive leaves can improve meat oxidative stability.
• Supplementation of both by-products did not affect the animals' performance.

## Introduction

Technological innovation applied to the reuse of agri-industrial by-products and residues in the feed sector is an advantageous situation from many aspects, including recovering the nutritional value of the by-products and improving the functionality and nutraceutical properties of diet. By-products are cheaper than grains and can reduce the ration cost. There are by-products rich in protein (corn gluten, meal extracted from vegetable oils, etc.), rich in digestible fibers (beet pulp, citrus pulp, etc.), rich in sugars and starch (meal and other by-products of the milling industry) that can be used in the formulation of complementary feeds. Moreover, some by-products are used and included in animal rations due to the particular concentration of bioactive compounds since, in addition to providing nutritional value, they play the role of functional supplement with particular beneficial effects on health or metabolism. In this case, the by-products are used similarly to feed additives and improve the health of the animal and the quality of the products; they can also *i)* favor the organoleptic qualities of the diets, *ii)* provide antioxidants, immune system stimulants, and digestive parasite inhibitors, *iii)* improve the quality of the products from the microbiological point of view and the stability of the lipids or the conservation and duration (Santana-Méridas et al., 2012; Valenti et al., 2019; Scerra et al., 2022a).

In our recent investigations (Scerra et al., 2022a), we demonstrated that supplementing the diet of growing pigs with ensiled bergamot pulp at a 15 % DM level increased the phenolic compounds and tocopherol levels in the diet. Similar findings have been reported by other researchers (Paiva-Martins et al., 2009; Botsoglou et al., 2012) using olive leaves, a by-product available in the same seasonal period as bergamot pulp. Although the incorporation of olive leaves improved the oxidative stability of raw pork (Paiva-Martins et al., 2009; Botsoglou et al., 2012), our studies showed that integrating bergamot pulp into the diet enhanced the shelf life of salami but not raw pork (Scerra et al., 2022a, 2022b), with slight differences on meat fatty acid composition in either case.

Considering that these by-products (i.e., bergamot pulp and olive leaves) are typically available simultaneously, it would be valuable to investigate the effects of their combined use on pork quality. Our hypothesis was that a higher inclusion of bergamot pulp — surpassing levels studied in previous research — and/or the synergetic effect with olive leaves could improve the pork shelf-life. Therefore, the aim of the present study was to assess the impacts of a 20 % DM inclusion of bergamot pulp in a pig's diet and the synergistic effects of combining bergamot pulp and olive leaves on animal performance and pork quality.

## Materials and methods

Thirty-six barrows of the Apulo-Calabrese breed obtained from a single farm were weighed (112.5 ± 7.40 kg initial body weight), randomly assigned to one of four treatments (9 animals per treatment), housed in individual pens and adapted for 7 days to the experimental treatments. Pigs received the experimental diets (Table 1) ad libitum for 100 days and had continuous access to water. The control group was fed a commercial concentrate-based diet. The other groups received a similar diet in which part of the cereals was replaced with 20 % (DM on the diet fed) of ensiled bergamot pulp (EBP group) or olive leaves (only olive leaves without olive pomace, OLL group) or a mixture (1:1) of ensiled bergamot pulp and olive leaves (BPOL group). Diet with olive leaves and/or bergamot pulp was a mixture of concentrate with the respective amount of by-product. In the diets of EBP, OLL and BPOL groups were integrated higher soybean meal content than the control diet to avoid feeding diets with different crude protein concentrations. The ingredients and chemical composition of the experimental diets are presented in Table 1.

**Table 1:** Ingredients (% on DM basis) and chemical composition of the experimental diets.

	Control diet	EBP diet	OLL diet	BPOL diet
Barley	30	20	20	20
Maize	30	20	20	20
Oat	15	11	11	11
Soybean meal	7	11	11	11
Faba bean	15	15	15	15
Olive leaves	-	–	20	10
Bergamot pulp	-	20	–	10
Vitamin mineral premix^1^	3	3	3	3
*Chemical composition*				
Dry matter (DM) g/kg wet weight	884	884	886	885
Crude protein g/kg DM	141	139	140	140
Ether extract g/kg DM	30.7	29.4	27.7	28.7
Ash g/Kg DM	38.9	39.2	36.2	38.1
NDF g/Kg DM	411	446	441	443
Total phenolic compounds (g TAe^2^/kg DM)	1.57	6.70	4.99	5.84
Total tannin compounds (g TAe^2^/kg DM)	1.19	1.71	2.11	1.91
*Tocopherols (μg/g DM)*				
α-Tocopherol	1.02	24.6	28.2	26.4
γ-Tocopherol	5.16	5.41	5.32	5.40
*Fatty acids (g/*100 g *of total fatty acid)*				
C10:0	0.03	0.01	0.06	0.09
C12:0	0.04	0.02	0.04	0.06
C14:0	0.82	0.72	0.88	0.80
C16:0	30.0	30.9	30.2	30.5
C18:0	4.01	3.90	4.19	4.05
C18:1 *n*-9	38.6	33.7	40.0	36.9
C18:2 *n*-6	25.6	27.0	21.7	24.3
C18:3 *n*-3	0.42	3.26	1.45	2.36

1 The mineral vitamin premix consisted of vitamina *A* = 6750 UI; vitamin D3 = 1000UI; vitamin E 2 mg; vitamin B12 0.01 mg; vitamin B1 1 mg; folic acid 0.2 mg; D-pantotenic acid 5 mg; Co 0.05 mg; Mn 12.5 mg; Zn 15 mg; Mo 0.5 mg.

2 tannic acid equivalent.

The BP was supplied by a juice citrus industry (Bova, Bova Marina, RC) and then ensiled in the experimental farm, while OL were obtained at a local oil mill (Melia Trasformazioni, Monasterace, RC) during the olive oil production period (October–December), dried at 35 °C (in a ventilated oven) for three days, milled and stored at room temperature until mixed with experimental feeds.

All the pigs were fed twice daily (0800 and 1500 h). The amounts of feed offered and refused were recorded every day in order to measure dry matter intake (DMI). Pigs were weighed every 20 days from the beginning to the end of the experimental trial.

After 100 days from the beginning of the trial, the pigs were transported to a commercial abattoir (15 min from experimental farm) and immediately slaughtered (electrically stunned) according to EU welfare guidelines. The animals were slaughtered after 8 h of fasting. Twenty minutes after slaughtering, the carcasses were weighed and stored at 4 °C. After 24 h from each carcass the *longissimus thoracis et lumborum* muscle (LTL) was removed and prepared for the analyses.

### Feedstuff analysis and meat proximate analysis

Feed samples were analyzed for crude protein, crude fat, and ash (AOAC,1995, methods 984.13, 920.39, 942.05 respectively) and neutral detergent fiber (NDF, method proposed by Van Soest et al., 1991). In feed samples, total phenolic compounds (extracted in aqueous acetone) were determined using the Folin– Ciocalteu reagent (Makkar et al., 1993). The vitamins α-tocopherol and γ-tocopherol were evaluated trough methanol:acetone:petroleum ether (1:1:1, v:v:v) extraction (Rufino-Moya et al., 2020) and ultra-high performance liquid chromatography (UHPLC), using a Shimadzu UHPLC (Nexera, Shimadzu Corporation, Milan, Italy) equipped with a Zorbax ODS column (25 cm × 4.6 mm, 5 µm; Agilent Technologies, CA). A Shimadzu spectrofluorometric detector (RF-20AXS) was used to determine tocopherols (295 nm excitation wavelength and 330 nm emission wavelength). A Shimadzu photodiode array detector (PDA; SPD-M40) was used to analyze retinol (absorbance at 325 nm).

The software LabSolutions controlled the UHPLC system. 10 μl of sample with methanol as mobile phase (flow rate of 1.3 ml min^−1^) was injected.

The analytes were identified by comparison of the retention times with those of the pure standards.

Fatty acid composition was analyzed following the procedures used by Valenti et al. (2018). Following AOAC (1995) procedures, moisture, crude fat, ash, and crude protein (methods 950.46, 991.36, 920.153 and 984.13 respectively) were determined in LTL samples.

### Meat fatty acid and antioxidant vitamins determination

Following the procedures described by Folch et al. (1957) fatty acid composition was analyzed on total lipids extracted. Briefly, fat was extracted from 10 g of homogenized samples with chloroform/methanol (1:1, v/v), then 50 mg of lipids was methylated adding 2 ml of hexane and 1 ml of 0.5 N sodium methylated in methanol (I.U.P.A.C., 1987). C19:0 was used as an internal standard and samples were analyzed using a gas chromatograph (model TRACE GC; Thermo Finnigan, Milan, Italy) equipped with a 100 m high-polar fused silica capillary column (5 mm i.d., 0.25 μm film thickness; SP. 24056; Supelco Inc., Bellefonte, PA). Gas-chromatography conditions and identification of FAME was performed as described by Scerra et al. (2022a). Fatty acids were quantified as mg/100 g of meat.

Antioxidant vitamins and cholesterol were determined in muscle following the procedures described by Natalello et al. (2023). Briefly, at 0.5 mg of meat sample were added 200 mg of L-ascorbic acid and 7.5 ml of 10 % KOH in ethanol:water (1:1) and saponified at room temperature overnight.

Them, 5 ml of 9:1 hexane:ethyl acetate with 25 mg/L of BHT was added. Subsequently, the tubes were centrifuged (at 10 °C, 5 min, 2000 × g). Supernatants were removed under nitrogen flow, dissolved in 1 ml of methanol, vortexed and filtered into a vial for UHPLC. Cholesterol was detected by absorbance wavelength at 220 nm. Chromatographic conditions were as described before for feed samples.

### Lipid oxidation and colour measurements

Three raw meat slices (2 cm thick) from each LTL samples were used to study the oxidative stability during 7 days of aerobic refrigerated storage. The slices were overwrapped with plastic wrap and stored in the dark at 4 °C. Colour parameters and lipid oxidation extent were determined after 2 h (for blooming), 3 and 7 days of storage. The colour descriptors L*, a*, b*, C* and hue angle (H*) were measured using a CR300 colour-meter (Minolta Co. Ltd. Osaka, Japan; aperture 6 mm, illuminant A and 10° standard observer).

Two more 2 cm thick meat slices were used to evaluate lipid oxidation in cooked meat, where the slices were vacuum-packaged and immersed in a 75 °C water bath for 30 min. One cooked slice was immediately used for lipid oxidation determination, whereas the other one was stored at 4 °C in the dark, measuring lipid oxidation after 2 days.

To study lipid oxidation in fresh and cooked meat during storage, thiobarbituric reactive substances (TBARS) were assessed (Siu & Draper, 1978). In brief, 2.5 g of samples (in 12.5 ml of distilled water) were mixed with 12.5 ml of 10 % trichloroacetic acid and, homogenized and filtered. Then, 4 ml of clear filtrate was mixed with 1 ml of 0.06 M aqueous thiobarbituric acid and incubated in a water bath at 80 °C for 90 min. Then, absorbance of the solution was measured at 532 nm using a UV-1800 spectrophotometer (Shimadzu Corporation, Milan, Italy). The assay was calibrated as reported in a previous paper (Scerra et al., 2022a).

### Statistical analysis

Individual pig was considered as experimental unit. All data were analyzed considering the dietary treatment as the fixed factor, using a GLM (general linear model) procedure. Data of colour descriptors and TBARS were analyzed using a GLM procedure for repeated measures, where the individual animal was included as a random factor, while the fixed factors in the model were the diet, the storage time and their interaction.

Multiple comparisons of the means were assessed using Tukey's test. The software used for statistical analyses was Minitab 14 (Minitab Inc, State College, PA).

## Results

As shown in Table 2, the administration of the four different diets to the pigs involved in the experimental trial did not lead to different final weights of the animals of the different groups (*P* = 0.449; Table 2). Also, other performance parameters, such as dry matter intake (*P* = 0.421; DMI), feed conversion ratio (*P* = 0.214; FCR), average daily gain (*P* = 0.586; ADG) and carcass weight (*P* = 0.823), were not influenced.

**Table 2:** Effect of dietary treatment on growth performance and chemical composition of *longissimus thoracis et lumborum* muscle (g/100 g wet weight).

	Dietary treatment				SEM^5^	*P* value
	Control	EBP	OLL	BPOL		
Final BW^1^, kg	146	144	142	145	4.740	0.449
Carcass weight, kg	122	119	120	121	4.280	0.823
Carcass yield,	83.5	82.4	84.3	83.4	3.567	0.621
total DMI^2^, kg/d	3.53	3.50	3.55	3.53	0.154	0.421
ADG^3^, g/d	340	320	300	330	24.20	0.586
FCR^4^, g (DMI^2^/g ADG^3^)	10.4	10.9	11.8	10.7	0.423	0.214
*Tocopherols and Cholesterol (µg/g muscle)*						
α-Tocopherol	2.28^b^	3.14^a^	3.23^a^	3.13^a^	0.184	0.019
γ-Tocopherol	0.30	0.28	0.25	0.28	0.022	0.112
Cholesterol	2.57	2.59	2.64	2.55	0.012	0.063
*Chemical composition*						
Moisture	73.6	74.0	73.7	74.1	0.187	0.781
Crude protein	22	21.6	21.3	21.5	0.173	0.603
Ether extract	2.11	1.98	2.25	2.08	0.140	0.520
Ash	1.14	1.14	1.19	1.17	0.112	0.506

1 BW = Body weight.

2 DMI = dry matter intake;^3^ADG = average daily gain;^4^FCR = feed conversion ratio;^5^SEM = standard error of means.

Regarding the chemical composition of meat (Table 2), no significant differences between groups were found for moisture, crude protein, ether extract and ash (*P* > 0.05). Vitamin E was mainly represented by α-tocopherol and its concentration in meat was affected by supplementing bergamot pulp and olive leaves, resulting higher (*P* < 0.019) in meat from EBP, OLL and EBOL groups than in meat from Control group.

In Table 3 are reported the effects of dietary treatments on the fatty acid composition of meat. In the present study, the dietary administration of by-products did not influence the accumulation of IMF (*P* > 0.05) in meat. The total of saturated fatty acids (SFA), monounsaturated fatty acids (MUFA) and polyunsaturated fatty acids (PUFA) were not different among groups (*P* > 0.05). As for individual fatty acids, no differences were observed among groups.

**Table 3:** Effect of the dietary treatments on fatty acid composition of *longissimus thoracis et lumborum* muscle (mg/100 g of muscle).

Item	Dietary Treatment					
	Control	EBP	OLL	BPOL	SEM	*P* value
intramuscular fat, mg/100 g of muscle	1914	1949	1907	1885	195	0.092
C12:0	0.89	0.93	0.82	1.07	0.155	0.757
C14:0	18.4	20.5	17.1	20.1	2.440	0.249
C15:0	0.30	0.34	0.37	0.42	0.113	0.131
C16:0	310	350	304	335	40.8	0.195
C16:1 *trans-*9	0.23	0.37	0.41	0.62	0.085	0.459
C17:0 *anteiso*	4.34	6.39	4.76	6.32	0.928	0.162
C16:1 *cis-9*	42.2	43.1	36.3	41.7	4.670	0.335
C 17:0	3.40	5.50	4.23	5.32	0.893	0.131
C17:1 *cis-10*	2.09	3.76	2.17	3.38	0.628	0.092
C18:0	155	182	154	180	21.4	0.167
C18:1 *trans-11* VA^1^	4.55	4.72	4.52	4.68	0.536	0.313
C18:1 *cis-9*	565	654	656	635	76.3	0.229
C18:1 *cis-11*	71.7	78.6	67.6	75.1	8.18	0.246
C18:2 *cis-9. cis-12* LA^1^	146	187	153	174	21.6	0.196
C 20:1 *cis11*	12.7	15.9	12.3	14.0	2.17	0.159
C18:3 *n-3* ALA^1^	4.90	7.31	5.24	7.02	0.891	0.073
C20:2 *n-6*	4.31	5.48	4.28	5.25	0.888	0.249
C20:3 *n-6*	1.02	0.92	1.64	1.38	0.159	0.502
C20:4 *n-6*	20.2	20.5	22.3	21.4	0.613	0.736
C20:5 *n-3* EPA^1^	2.43	3.22	2.73	2.95	0.211	0.848
C22:6 *n-3* DHA^1^	1.29	1.98	1.73	1.47	0.196	0.327
∑ SFA^1^	487	559	480	541	66.5	0.186
∑ MUFA^1^	698	800	779	774	92.2	0.230
∑ PUFA^1^	180	226	191	213	24.1	0.195
∑ *n-3*	8.63	12.5	9.70	11.4	1.19	0.082
∑ *n-6*	171	213	181	202	22.8	0.197
∑ PUFA^1^/∑ SFA^1^	0.37	0.40	0.40	0.39	0.016	0.328
*n-6/n-3*	19.9	17.1	18.6	17.6	0.601	0.621

1 VA: Vaccenic acid; LA: linoleic acid; ALA: α-linolenic acid; EPA: Eicosapentaenoic acid; DHA: docosahexaenoic acid; SFA: saturated fatty acids; MUFA: monounsaturated fatty acids; PUFA: polyunsaturated fatty acids.

**Fig. 1:**
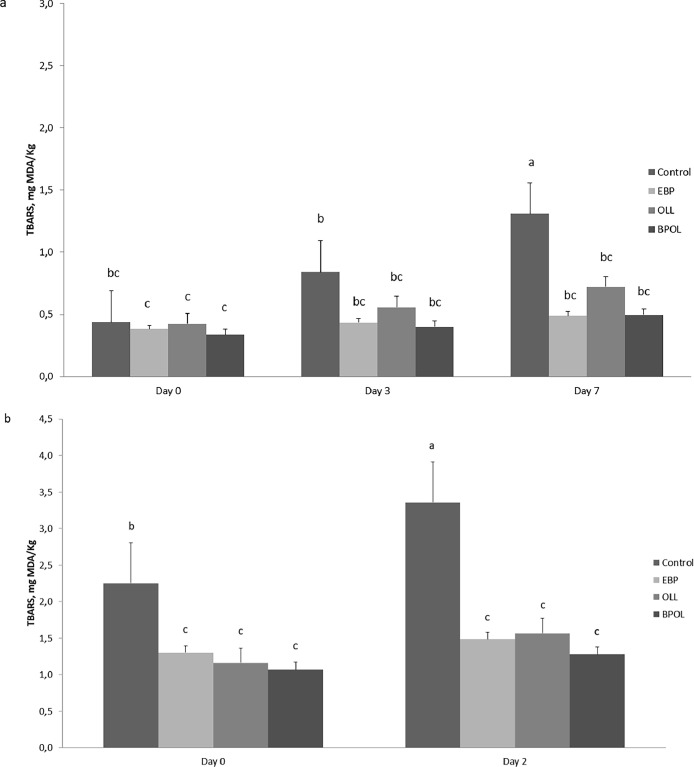
Effect of the dietary treatment and time of storage on the oxidative stability of longissimus thoracis et lumborum muscle, raw (a) and cooked (b). Treatments were: Control, concentrate-based diet; EBP, concentrate and ensiled bergamot pulp at the level of 20 % DM on the diet fed; OLL, concentrate and olive leaves at the level of 20 % DM on the diet fed; BPOL, concentrate and a mixture 1:1 of ensiled bergamot pulp and olive leaves at the level of 20 % DM on the diet fed. Interactive effect of the dietary treatment (Control, EBP, OLL and BPOL) and time of storage on the TBARS values measured in raw and cooked slices over aerobic storage at 4 °C. Values presented are the estimated least squares means and standard error bars. ^a,b,c,d^Values with different superscripts are significantly different (*P* < 0.05).

Fig. 1 shows the TBARS values measured during monitoring days. The TBARS values increased over storage duration (*P* < 0.05) only in raw and cooked meat from control group, remaining constant in meat from EBP, OLL and EBOL groups during all monitoring days, with a value of diet × time interaction highly significant (*P* < 0.001).

Regarding colour descriptors (Table 4), the redness (a*), lightness (L*) and yellowness (b*) were not affected by storage time and dietary treatment. Instead, hue angle (H*) values were affected by storage time (*P* < 0.001), with progressively decreasing values.

**Table 4:** Effect of the dietary treatments and time of refrigerated storage on meat colour stability of *longissimus thoracis et lumborum* muscle.

	Dietary treatment				Time^2^			SEM	*P* values		
	Control	EBP	OLL	BPOL	0	1	2		Diet	Time	Diet × Time
L* values^1^	46.2	48.1	47.0	47.8	47.9	46.4	47.6	0.363	0.244	0.221	0.997
a* values^1^	7.1	7.0	7.3	7.9	7.3	7.2	7.7	0.145	0.099	0.434	0.935
b* values^1^	6.1	5.8	5.8	6.4	6.5	5.9	5.6	0.138	0.110	0.097	0.939
C* values^1^	9.4	9.2	9.4	10.2	9.8	9.3	9.4	0.176	0.128	0.259	0.997
H* values^1^	41.0	39.5	38.3	39.2	42.3^x^	39.5^y^	36.5^z^	0.576	0.538	0.001	0.226

x.y.z Within row, different superscripts indicate differences between days of storage (*P* < 0.05) tested using the Tukey's adjustment for multiple comparisons.

1 L* = lightness; a* = redness; b* = yellowness; C* = Chrome; H* = hue angle, measured in degrees.

2 Times 0, 1, 2 = days 0, 3, 7 for raw meat at 4 °C under aerobic conditions (meat slices).

## Discussion

Scarce recent data are available in literature on the effects of the use of OL on animal performance and meat quality (Paiva-Martins et al., 2009), while interesting results have recently been reported for the use of ensiled BP (Scerra et al., 2022a) or exhausted bergamot by-product (Scerra et al., 2022b) in pig feeding from our experimental trials. However, no studies have been conducted to evaluate the effects of the simultaneous use of BP and OL - which are usually available on the same season - in pig diets on animal performance and meat quality.

The administration of the four different diets to the pigs involved in the experimental trial did not lead to a variation of performance parameters among the experimental groups. Similar results were observed in our previous studies (Scerra et al., 2022a) in which pigs were fed a diet integrated with ensiled bergamot pulp and in a trial where pigs received a diet integrated with 10 g/kg of OL (Botsoglou et al., 2012). Conversely, dietary OL negatively affected daily gain and final body weight of pigs in a study carried out by Paiva-Martins et al. (2009), where the authors underline that the incorporation of 5 % or 10 % of olive leaves in diets for growing pigs increased the levels of dietary fibre and led to impaired growth rates and feed:gain ratio, as observed by other authors in rabbits (García et al., 1999). However, Paiva-Martins et al. (2009) also observed a decrease of DMI in the animals supplemented with OL and this probably negatively affected final body weight. In the present study, the lack of effects on animal performances could be partially explained in two different manners. (*i*) EBP, OLL and BPOL diets had a higher in soybean meal content than the control diet in order to obtain isoproteic treatments. This may have led to an improved amino acid profile and, in turn, a positive influence on the growth rates of the animals in these experimental groups. (*ii*) This result may also have been influenced by the breed used for the experimental trial. The Apulo-Calabrese pig is a native Calabria breed that, as reported by Micari et al. (2009) and Pugliese and Sirtori (2012) has a high adaptability to different production systems compared to cosmopolitan breeds. As reported above, even the dietary supplementation of 20 % of a mixture (1:1) of BP and OL did not influence the growth performance of the pigs. Considering the results obtained using the by-products individually, this result was predictable.

The partial replacement of cereals with BP or OL or a mix of both by-products did not lead to changes in meat fatty acid composition (Table 3). Considering the higher percentage of α-linolenic acid in the diets supplemented with the examined by-products, we expected also in the meat from the groups that received these diets a higher level of α-linolenic acid compared to the control group, at least from the groups whose diets included 15 % BP, as observed in our previous experimental study (Scerra et al., 2022a). Actually, an increasing trend of α-linolenic acid was observed in meat from EBP and BPOL groups compared to the control group, as well as in total n-3 fatty acids. No significant differences in fatty acid composition of meat were observed by Paiva-Martins et al. (2009) integrating 10 % of olive leaves in the diet of pigs.

Dietary integration of by-products tested in this trial led to greater oxidative stability of the meat during the days of storage in both fresh and cooked meat (Fig. 1). In EBP, OLL and BPOL groups the TBARS values not increased during storage in fresh and cooked meat, while increased in meat from the control group. Specifically, in raw meat from animals fed the control diet lipid oxidation increased significantly after 7 days of refrigerated storage (*P* < 0.001), while TBARS values were remained stable throughout the monitoring period for the other three experimental groups. As in raw meat, in cooked meat TBARS values were higher (*P* < 0.01) in the control group than in the EBP, OLL and BPOL groups after 2 days of storage. No significant differences were observed between the groups that received BP or OL or a mixture of both by-products. Similar results were observed by Paiva-Martins et al. (2009) in pigs fed a diet supplemented with dried olive leaf powder at 5 % and 10 %, where after 8 days of storage at 4 °C the peroxide and conjugated dienes values of muscles were higher in pigs fed a conventional diet. In contrast, no significant differences were observed in our previous study (Scerra et al., 2022a) in fresh and cooked meat obtained from pigs fed diets that included BP at the level of 15 % than meat from pigs fed a conventional diet. However, in this study (Scerra et al., 2022a), in the salami (a product that has been subjected to pro-oxidation conditions) obtained from animals supplemented with BP, the TBARS values were lower already after 2 days of storage than in salami from animals fed a concentrate-based diet.

Data reported by our recent study and by other authors highlight that the integration of BP (Scerra et al., 2022a) or OL (Paiva-Martins et al., 2009, 2014), into the diet increased the level of antioxidant compounds. The improvement of oxidative stability in meat products following the integration of BP into the diet was attributed to the higher amount of phenolic compounds (Scerra et al., 2022a), considering that no vitamins differences were observed in salami from the experimental groups, whereas Paiva-Martin et al. (2009) attributed the improvement in the oxidative stability of meat to the greater amount of vitamin E provided by the diets that included OL. In the present trial, meat from pigs that received EBP, OLL and BPOL diets showed a higher (*P* < 0.01) amount of α-tocopherol than meat from pigs of the control group. The level of α-tocopherol in BP and OL was similar (data not shown, 89 and 100 ug/g DM, respectively) and their inclusion as cereals replacement increased the ingestion of tocopherol in EBP, OLL and BPOL diets, thus leading to higher levels of this important antioxidant molecule in the meat of the animals from the groups fed the tested by-products compared to the meat from the control group.

The latter data probably influenced the different trends of lipid oxidation during the 7 days of monitoring between the EBP, OLL and BPOL groups and the control one. Furthermore, this data could also have been influenced by the level of phenolic compounds in the diets, which resulted 3–4 times higher in diets that included BP or OL or both compared to the control diet. The presence of phenolic compounds in the BP and OL could have indirectly preserved vitamin E during digestion thanks to their antioxidant activity, leading to greater absorption of tocopherols by the animals. The TBARS value at day 0 already exceeds the threshold value of 2 mg MDA/kg of meat (maximum level for positive sensory perception; Campo et al., 2006), in cooked meat from animals of the control group, while it remains below the threshold value for the entire monitoring period for the other 3 experimental groups.

Regarding meat colour, all colour descriptors were not affected by storage time or dietary treatment except the hue angle (H*), which was affected by storage time. These results were not expected considering that, following the typical meat discoloration pattern (Luciano et al., 2019), the descriptor L*, b*, and hue angle should increase while a* should decrease during storage. Some studies (Alderton et al., 2003; Faustman et al., 2010) reported that the oxidation of myoglobin, the main pigment of meat, is promoted by fatty acid oxidation. The TBARS values found in raw meat from the control group after 7 days of monitoring, although higher than those observed in the EBP, OLL and BPOL groups, remain well below the value considered a maximum level for positive sensory perception (Campo et al., 2006), indicating that in general the meat from all experimental groups showed good resistance against oxidative phenomena.

## Conclusion

In this study, the partial replacement of cereals with 20 % DM on the diet fed of ensiled bergamot pulp and olive leaves, either alone or in combination, in the pig's diet, led to greater oxidative stability of the meat during the days of storage in both fresh and cooked meat. These data were likely influenced by the higher levels of α-tocopherol in meat from pigs that received these by products, either alone or in combination, than in meat from pigs fed a commercial concentrate. Furthermore, the level of phenolic compounds in the diets, higher in diets that included ensiled bergamot pulp and/or olive leaves, may also have influenced the improvement of oxidative stability. Conversely, no effect of diet was observed on animal performance and on fatty acid composition of meat.

Further studies are needed to evaluate bergamot pulp and olive leaves at different proportions of inclusion in the pigs’ ration and with difference genetic types (e.g., cosmopolitan breeds) in order to make these two new feeds commonly employed in all pig farms present in the same production areas as the by-products.

## Availability of data

None of the data were deposited in an official repository. The data that support the study findings are available from the authors upon request.

## Declaration of generative AI and AI in scientific writing

The authors did not use any artificial intelligence-assisted technologies in the writing process.

## Ethics statement

This experiment was approved by the Animal Welfare Committee of the University of Reggio Calabria (No. 8937).

## CRediT authorship contribution statement

**Manuel Scerra:** Writing – review & editing, Writing – original draft, Methodology, Conceptualization. **Francesco Foti:** Writing – review & editing, Formal analysis. **Pasquale Caparra:** Writing – review & editing. **Caterina Cilione:** Writing – review & editing, Formal analysis. **Matteo Bognanno:** Writing – review & editing. **Fortugno Paolo:** Writing – review & editing, Formal analysis. **De Caria Paolo:** Writing – review & editing, Formal analysis. **Antonio Natalello:** Writing – review & editing, Investigation, Formal analysis. **Martino Musati:** Writing – review & editing, Formal analysis. **Luigi Chies:** Writing – review & editing.

## Declaration of competing interest

The authors declare that they have no known competing financial interests or personal relationships that could have appeared to influence the work reported in this paper.
